# The synthetic xylulose-1 phosphate pathway increases production of glycolic acid from xylose-rich sugar mixtures

**DOI:** 10.1186/s13068-016-0610-2

**Published:** 2016-09-20

**Authors:** Ceren Alkim, Debora Trichez, Yvan Cam, Lucie Spina, Jean Marie François, Thomas Walther

**Affiliations:** 1 LISBP, CNRS, INRA, INSA, Université de Toulouse, 135 Avenue de Rangueil, 31077 Toulouse, France; 2 TWB, 3 rue Ariane, 31520 Ramonville-St. Agne, France

**Keywords:** *Escherichia coli*, Glycolic acid, Synthetic pathway, Glucose, Xylose

## Abstract

**Background:**

Glycolic acid (GA) is a two-carbon hydroxyacid with applications in the cosmetic, textile, and medical industry. Microbial GA production from all sugars can be achieved by engineering the natural glyoxylate shunt. The synthetic (d)-xylulose-1 phosphate (X1P) pathway provides a complementary route to produce GA from (d)-xylose. The simultaneous operation of the X1P and glyoxylate pathways increases the theoretical GA yield from xylose by 20 %, which may strongly improve GA production from hemicellulosic hydrolysates.

**Results:**

We herein describe the construction of an *E. coli* strain that produces GA via the glyoxylate pathway at a yield of 0.31 , 0.29 , and 0.37 g/g from glucose, xylose, or a mixture of glucose and xylose (mass ratio: 33:66 %), respectively. When the X1P pathway operates in addition to the glyoxylate pathway, the GA yields on the three substrates are, respectively, 0.39 , 0.43 , and 0.47 g/g. Upon constitutive expression of the sugar permease GalP, the GA yield of the strain which simultaneously operates the glyoxylate and X1P pathways further increases to 0.63 g/g when growing on the glucose/xylose mixture. Under these conditions, the GA yield on the xylose fraction of the sugar mixture reaches 0.75 g/g, which is the highest yield reported to date.

**Conclusions:**

These results demonstrate that the synthetic X1P pathway has a very strong potential to improve GA production from xylose-rich hemicellulosic hydrolysates.

## Background

Glycolic acid (GA) is a two-carbon hydroxycarboxylic acid of considerable industrial interest. It is used as a tanning, peeling, and cleaning agent in the cosmetic and textile industry [1–3]. GA can be polymerized to produce biodegradable poly-glycolic acid (PGA) which is used as a packaging material for food and beverages [4]. Co-polymers of PGA and poly-lactic acid are used as absorbable suture and implant matrices [5, 6]. The market volume of GA continues to grow substantially and was reported to be 40 kilotons in 2014 [7].

At the industrial scale, GA is produced from fossil resources by treating formaldehyde with carbon monoxide [8], or by treating chloroacetic acid with sodium hydroxide [2]. Growing concerns about the future availability of fossil resources and the environmental impact of their use [9, 10] have increased the interest in microbial production of GA. Until recently, sugar-based biosynthesis of GA was exclusively achieved by engineering the natural glyoxylate pathways in bacteria or yeast. GA production was accomplished by overexpressing isocitrate lyase and glyoxylate reductase enzymes, by deleting glyoxylate consuming reactions, and in some cases by attenuating isocitrate dehydrogenase activity ([11–15] and Table 1). The best results were obtained with an optimized *Escherichia coli* strain which produced 56 g/l GA at a yield of 0.52 g/g in a fed-batch reactor using glucose as the carbon source ([11], Table 1).

**Table 1:** Production of glycolic acid (GA) by different engineered microorganisms

Microorganism	Strain characteristics	Experimental conditions	Final GA conc. [g/L]	GA yield [g/g]	References
*Escherichia coli*	Engineered glyoxylate shunt Reduced expression of *icd* by weak promoter Deletion of *aceB*, *gcl*, *glcDEFGB*, *aldA*, *iclR*, *edd*-*eda*, *poxB*, *ackA* + *pta*, *ldhA*, *mgsA*, *arcA* Overexpression of *ghrA* and *aceA*	Shake flask Mineral medium + 10 g/l glucose	5.14	0.52	[12]
	Engineered glyoxylate shunt Reduced expression of *icd* by weak promoter Deletion of *aceB*, *gcl*, *glcDEFGB*, *aldA*, *iclR*, *edd*-*eda*, *poxB*, *ackA* + *pta*, *arcA* Overexpression of *ghrA* and *aceA*	Fed-batch fermentation Mineral medium + glucose	52.2	0.38	
*Kluyveromyces lactis*	Engineered glyoxylate shunt Deletion of *MLS1*, *IDP2* Overexpression of *GLYR1*	Fed batch fermentation Mineral medium with xylose and ethanol at mass ratio of 1:35	15	nr	[13]
*Corynebacterium glutamicum*	Engineered glyoxylate shunt Reduced expression of *icd* Deletion of *aceB* Overexpression of *ghrA*	Shake flask CGXII minimal medium (10.8 g/l glucose + 10.6 g/l acetate)	5.30	0.18	[15]
*E. coli*	Synthetic (d)-xylulose-1P pathway Expression of *khkC*, *aldoB*, *aldA* Deletion of *xylB*, *glcD*	Shake flask Mineral medium + 10 g/l xylose	4.30	0.46	[16]
*E. coli*	Engineered glyoxylate shunt Deletion of *aceB, glcB* Overexpression of *ghrA, aceA, aceK* Evolved strain	Shake flask Mineral medium + 8 g/l glucose	2.66	0.33	[11]
		Batch bioreactor Mineral medium + 19 g/l glucose	8.96	0.48	
		Fed batch bioreactor Mineral medium + glucose	56.44	0.52	
*E. coli*	Synthetic xylulose epimerase pathway Expression of *Pc*-*dte, fucK, fucA, aldA* Deletion of *glcD*, *xylB*	Test tube Mineral medium + 10 g/l xylose	3.43	0.46	[14]
		Batch bioreactor Mineral medium + >100 g/l xylose	44.00	0.44	
	Synthetic xylulose epimerase pathway+engineered glyoxylate shunt Expression of *Pc*-*dte, fucK, fucA, aldA* Deletion of *glcD*, *xylB, aceB, glcB, gcl* Overexpression of *ghrA*, *aceA* and *aceK*	Batch bioreactor Mineral medium + 65 g/l xylose	40.00	0.63	
*E. coli*	Engineered glyoxylate shunt	Shake flask Mineral medium + 10 g/l glucose (2 g/l tryptone, 1 g/l yeast extract)	2.64	0.31	This study
	Synthetic (d)-xylulose-1P pathway + engineered glyoxylate shunt Expression of *khkC, aldoB, aldA* Deletion of *aceB*, *gcl*, *glcDEFGB*, *iclR*, *edd*-*eda*, *arcA, icd, xylB* Overexpression of *ghrA* and *aceA*	Shake flask Mineral medium + 10 g/l xylose (2 g/l tryptone, 1 g/l yeast extract)	2.24	0.43	
	Synthetic (d)-xylulose-1P pathway + engineered glyoxylate shunt Expression of *khkC, aldoB, aldA* Deletion of *aceB*, *gcl*, *glcDEFGB*, *iclR*, *edd*-*eda*, *arcA, icd, xylB* Overexpression of *ghrA* and *aceA* *galP*expressed from constitutive promoter proD	Shake flask Mineral medium + 2,5 g/l glucose and 5 g/l xylose (2 g/l tryptone, 1 g/l yeast extract)	3.73	0.63 (0.75)*	

* Estimated yield on the xylose fraction of the sugar mixture

Glycolic acid production through the glyoxylate pathway inevitably requires the decarboxylation of pyruvate to provide the Krebs cycle substrate acetyl-CoA. Thus, the maximum yields for the glyoxylate pathway-dependent biosynthesis of GA from glucose or xylose are limited to 2 mol/mol (0.84 g/g) and 1.66 mol/mol (0.84 g/g), respectively. Recently, two synthetic pathways have been proposed that provide an improved stoichiometry for the synthesis of GA from (d)-xylose thereby increasing the maximum GA yield to 2 mol/mol (1 g/g). Both pathways employ a carbon-conserving asymmetric aldolase cleavage of a C5 sugar that produces the C2 compound glycolaldehyde which is a direct precursor of GA, and the C3 compound DHAP [14, 16]. Our group has developed the reaction sequence that employs xylulose-1 kinase, xylulose-1-phosphate (X1P) aldolase, and glycolaldehyde dehydrogenase activities [16]. These activities were provided by expressing the genes encoding human ketohexokinase C (*khkC*), aldolase B (*aldoB*), and endogenous aldehyde dehydrogenase (*aldA*), respectively. We have termed this reaction sequence (d)-xylulose-1-phosphate pathway, and demonstrated the production of GA from pure xylose at a yield of 0.46 g/g corresponding to 94 % of the maximum yield of this pathway ([16], Table 1). Pereira and colleagues (2016) used a (d)-xylulose epimerase to transform (d)-xylulose into (d)-ribulose which was further converted to glycolaldehyde and DHAP by the consecutive action of (d)-ribulose-1 kinase and (d)-ribulose-1 aldolase. They demonstrated GA production from pure (d)-xylose during simultaneous operation of the glyoxylate and xylulose epimerase pathways, and achieved a GA yield of 0.63 g/g ([14], Table 1).

**Fig. 1:**
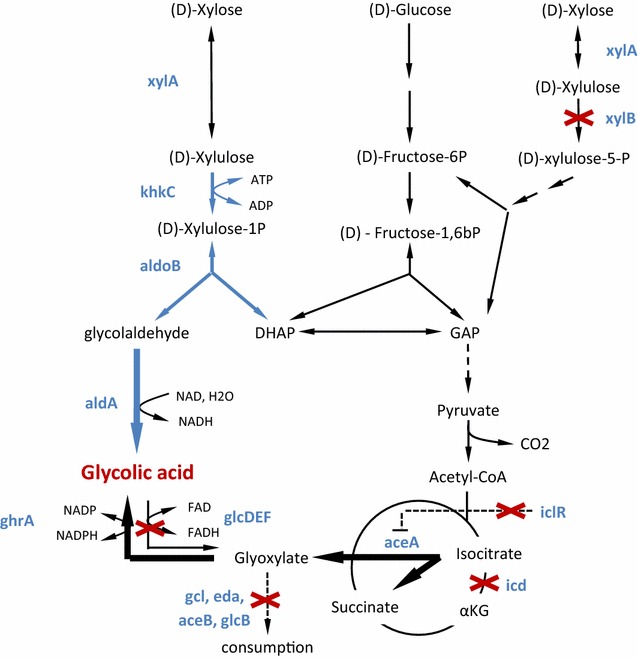
Synthetic (*blue*) (D)-xylulose-1 phosphate (X1P) and natural (*black*) pathways for the production of glycolic acid from (d)-xylose and (d)-glucose. Relevant genes are depicted in *blue* next to the reactions that they encode. *Bold black arrows* indicate overexpression of the activity. *DHAP* dihydroxyacetone phosphate, *GAP* glyceraldehyde-3P, *α-KG * α-ketoglutarate)

However, the utilization of pure xylose is an unrealistic scenario for the industrial production of GA. Although (d)-xylose can account for up to 80 % of the sugar fraction of hemicellulosic hydrolysates, the glucose content in these feedstocks is still substantial and reaches up to 35 % depending on raw material and hydrolyzation method [17]. Therefore, we investigated the potential of the synthetic X1P pathway to increase GA production on a synthetic sugar mixture that contained xylose and glucose at a ratio of 66 and 33 % which we considered representative for a large panel of hemicellulosic hydrolysates. We engineered an *E. coli* strain to facilitate GA production through the glyoxylate pathway alone or in combination with the X1P pathway (Fig. 1). When GA was exclusively synthesized via the glyoxylate shunt, the strain produced GA at a yield of 0.37 g/g from the sugar mixture. Upon the additional operation of the X1P pathway, the GA yield increased to 0.47 g/g, and reached 0.63 g/g when the broad-range sugar permease GalP was additionally overexpressed from a constitutive promoter. Under these conditions, the GA yield on the xylose fraction of the sugar mixture reached 0.75 g/g, which corresponds to 75 % of the theoretical maximum yield.

## Methods

### Strains and plasmid construction

*Escherichia coli* K-12 MG1655 (ATCC 47076) was used as the parental strain for all strain constructions in this study. The constructed strains are listed in Table 2. Gene deletions were introduced either by homologous recombination using the λ Red recombinase system [18], in the case of *glcDEFGB*, *edd*-*eda*, and *iclR*, or by the phage transduction method [19], in the case of *aceB*, *gcl*, *arcA*, *icd* and *xylB*. Gene deletion cassettes were amplified from pKD3 or pKD4 plasmids (Table 3) that have a chloramphenicol or a kanamycin resistance marker, respectively, using primers with 50 bp homologies to the target locus. The deletion cassettes were purified using a PCR purification kit (Thermo Scientific) and transformed into the target strains using a standard protocol [20]. Cell lysates for phage transductions were prepared from single-gene deletion mutants of the Keio strain collection [21].

**Table 2:** *Escherichia coli* strains used in this study

Strain name	Genotype	Reference
MG1655	F^−^ λ^−^ilvG-rfb-50 rph-1	ATCC 47076
NEB5-α	*fhuA2 Δ(argF*-*lacZ)U169 phoA glnV44 Φ80Δ (lacZ)M15 gyrA96 recA1 relA1 endA1 thi*-*1 hsdR17*	NEB
JW3536-2	F-*, Δ(araD*-*araB)567, ΔlacZ4787(::rrnB*-*3), λ*-*, rph*-*1, Δ(rhaD*-*rhaB)568, hsdR514 ΔxylB747::kan*	[21]
JW3974-1	F-*, Δ(araD*-*araB)567, ΔlacZ4787(::rrnB*-*3), λ*-*, rph*-*1, Δ(rhaD*-*rhaB)568, hsdR514 ΔaceB781::kan*,	[21]
JW0495-1	F-*, Δ(araD*-*araB)567, ΔlacZ4787(::rrnB*-*3), λ*-*, rph*-*1, Δ(rhaD*-*rhaB)568, hsdR514 Δgcl*-*790::kan*	[21]
JW4364-1	F-*, Δ(araD*-*araB)567, ΔlacZ4787(::rrnB*-*3), λ*-*, rph*-*1, Δ(rhaD*-*rhaB)568, hsdR514 ΔarcA726::kan*	[21]
JW1122-2	F-*, Δ(araD*-*araB)567, ΔlacZ4787(::rrnB*-*3), λ*-*, rph*-*1, Δ(rhaD*-*rhaB)568, hsdR514 Δicd*-*724::kan*	[21]
Pen804	*ΔaceB ΔglcDEFGB Δgcl Δedd*-*eda*	This study
Pen807	Pen804 containing pGS	This study
Pen847	Pen804 *ΔiclR::FRT* pGS	This study
Pen851	Pen847 *ΔarcA::FRT* pGS	This study
Pen1046	Pen851 *Δicd::FRT*	This study
Pen1042	Pen1046 containing pGS	This study
Pen1043	Pen1046 containing pX1P	This study
Pen1044	Pen1042 containing pX1P	This study
Pen1099	Pen1046 *galP* ^*proD*^ containing pGS and pX1P	This study
Pen1100	Pen1046 containing pACT3-empty	This study
Pen880	*ΔaceB ΔglcDEFGB Δgcl Δedd*-*eda ΔiclR ΔarcA Δicd ΔxylB*	This study
Pen1048	Pen880 containing pX1P	This study
Pen905	Pen1048 containing pGS	This study
Pen979	Pen880 *galP* ^*proD*^ containing pGS and pX1P	This study

**Table 3:** Plasmids used in this study

Name	Relevant characteristics	Reference
pCP20	*ori* pSC101, Amp^R^, plasmid expressing Flp recombinase to remove Kan cassette	[42]
pKD46	*ori* oriR101 w/repA101ts, Amp^R^, plasmid expressing λ Red recombinase genes	[18]
pKD3	*ori* R6 Kγ, Cm^R^, source for cat cassette	[18]
pKD4	*ori* R6 Kγ, Kan^R^, source for kan cassette	[18]
pACT3	*ori* p15A, Cm^R^	[23]
pEXT20	*ori* colE1, Amp^R^	[23]
pGS	pACT3 derivative carrying *ghrA* and *aceA* genes from *E. coli*	This study
pX1P	pEXT20 derivative carrying human genes *khk*-*c* and codon optimized *aldoB*, and *aldA* from *E. coli*	[16]

Expression of *galP* was rendered constitutive by replacing the natural chromosomal 5′-UTR of *galP* by the synthetic constitutive promoter proD [22]. The proD sequence was synthesized by Eurofins™. The kanamycine resistance cassette of the pKD4 plasmid and the synthetic promoter were individually amplified by Phusion polymerase (Biolabs) and fused by overlap extension PCR adding 50 bp flanking sequences that were homologous to the target locus. The resulting DNA fragment was used to replace the natural *galP* promoter by homologous recombination [18].

Plasmid constructions: *aceA* and *ghrA* genes were PCR amplified from *Escherichia coli* K-12 MG1655 genomic DNA using Phusion polymerase (Biolabs) with primers listed in Table 4. The DNA fragments were purified using a PCR purification kit (Thermo Scientific). The medium-copy pACT3 plasmid [23] was linearized with *Bam*HI and *Hind*III (BioLabs), and the DNA fragments and the linearized vector were recombined using the *In*-*Fusion*® HD Cloning Kit (Clontech). The resulting plasmid was named pGS. The construction of plasmid pXlP, which expresses the genes encoding for aldehyde dehydrogenase from *E. coli*, and human ketohexokinase C and aldolase B, respectively, was described previously [16]. The plasmids were transformed into different host strains alone or in combination. Strains and primers used in these studies are listed in Tables 2 and 3, respectively.

**Table 4:** Primers used in this study

Primer	Sequence 5′→ 3′
Gene deletions by homologous recombination	
glcDEFGB_fw	GCGTCTTGATGGCGCTTTACCCGATGTCGACCGCACATCGGTACTGATGGCACTGCGTGAGCATGTCCCTGGACTTGAGATCGTGTAGGCTGGAGCTGCTTC
glcDEFGB_rev	CGCGTAAACGCCAGGCGTGTAATAACGGTTCGGTATAGCCGTTTGGCTGTTTCACGCCGAGGAAGATTAAATCGCTGGCCATATGAATATCCTCCTTAG
edd eda_fw	CGCGCGAGACTCGCTCTGCTTATCTCGCCCGGATAGAACAAGCGAAAACTTCGACCGTTCATCGTTCGCAGTTGGCATGCGGGTGTAGGCTGGAGCTGCTTC
edd eda_rev	GCTTAGCGCCTTCTACAGCTTCACGCGCCAGCTTAGTAATGCGGTCGTAATCGCCCGCTTCCAGCGCATCTGCCGGAACCCATATGAATATCCTCCTTAG
iclR_fw	CGCACCCATTCCCGCGAAACGCGGCAGAAAACCCGCCGTTGCCACCGCACCAGCGACTGGACAGGTTCAGTCTTTAACGCGTGTAGGCTGGAGCTGCTTCG
iclR_rev	GCGCATTCCACCGTACGCCAGCGTCACTTCCTTCGCCGCTTTAATCACCATCGCGCCAAACTCGGTCACGCGGTCATCGGCATATGAATATCCTCCTTAG
Construction of synthetic galP promoter	
galP-KAN-fw	CCGCCCGCA CAATAACATCATTCTTCCTG ATCACGTTTCACCGCAGATTAGTGTAGGCTGGAGCTGCTTC
galP-KAN-rev	GATAGGGACGACGTGGTGTTAGCTGTGCATATGAATATCCTCCTTAG
galP-prom-fw	CACAGCTAACACCACGTCGT
galP-prom-rev	ACGTCATTGCCTTGTTTGACCGCCCCTGTTTTTTAGCGTCAGGCATATAATACCTCCTAAAGTTAAACAAAATTATTTGTAG
Prom_galP^proD^_veri_fw	GCTGGCCTTTTTCTTTTGGATAG
Prom_galP^proD^_veri_rev	ACCGATATCCAGGCCAAAGAG
Verification Primers for gene knockouts	
xylB_fw_2	GTTATCGGTAGCGATACCGGGCATTTT
xylB_rev_2	GGATCCTGAATTATCCCCCACCCGGTCAGGCA
aceB_verif_fw	CATGAATCCAACGCTGGATT
aceB_verif_rev	CGAGGCTGTTGATGTAGCC
fw glDEF	TCCCGGACCTCGTGCACAGGTA
glcB_verif_rev	CACACGCAGACGCAGAGTA
gcl_verif_fw	TGTAGGTCTGAATTGCATAG
gcl_verif_rev	CACGGGCATAACGAATCGCT
eda_verif_rev	CCTTCCTCGGACTTCCGGTT
eda_verif_rev	CCTTCCTCGGACTTCCGGTT
iclR_verif2_fw	TTTCACCGCAAATACCGCCG
iclR_verif2_rev	TGCAGCAATGTGTCGGCATAC
arcA_verif_fw	CCTGAGGGAAAGTACCCACG
arcA_verif_rev	GTTGTTGGGAACCAGTGTGC
icd-verif-fw	CGACCTGCTGCATAAACACC
icd-verif-rev	TGAACGCTAAGGTGATTGCA
Cloning of ghrA and aceA	
pACT ycdW-Fw	GAGCTCGGTACCCGG*GGATCC*AGGAGGCACACG***ATG***GATATCATCTTTTATCACC
Operon ycdW-Rev	TCATACGGTTCCTCCTTTAGTAGCCGCGTGCGCGGTCGACTT
Operon aceA-Fw	GCACGCGGCTACTAAAGGAGGAACCGT***ATG***AAAACCCGTACACAACAAATTG
pACT aceA-Rev	CTCATCCGCCAAAACAG*AAGCTT*TTAGAACTGCGATTCTTCAGTGGA

Restriction sites are italicized and the start codons are shown in bold–italics

### Media and cultivation conditions

Luria–Bertani (LB) medium [24] was used for preparations of precultures and genetic manipulations. Growth and production cultures were carried out in M9 minimal medium which contained (d)-glucose, (d)-xylose or a mixture of (d)-glucose/(d)-xylose. Carbon source concentrations of M9 minimal medium are indicated in the text. M9 minimal medium contained 18 g/l Na_2_HPO_4_ · 12 H_2_O, 3 g/l KH_2_PO_4_, 0.5 g/l NaCl, 2 g/l NH_4_Cl, 0.5 g/l MgSO_4_ · 7 H_2_O, 0.015 g/l CaCl_2_ · 2 H_2_O, 0.010 g/l FeCl_3_, 0.006 g/l Thiamine HCl, 0.4 mg/l NaEDTA · 2 H_2_O, 1.8 mg/LCoCl_2_ · 6 H_2_O, 1.8 mg/l ZnCl_2_SO_4_ · 7 H_2_O, 0.4 mg/L Na_2_MoO_4_ · 2 H_2_O, 0.1 mg/L H_3_BO_3_, 1.2 mg/L MnSO_4_ · H_2_O, 1.2 mg/L CuCl_2_ · 2 H_2_O. The medium was buffered at pH 7 by addition of 20 g/l MOPS (3-(*N*-morpholino) propanesulfonic acid) and sterilized by filtration (Merck Millipore ExpressPlus). 0.2 % ‘w/v’ tryptone and 0.1 % ‘w/v’ yeast extract were added to the M9 minimal medium from 5× sterile stock solutions to grow strains with an *icd* deletion. When required, ampicillin, kanamycin and chloramphenicol were added to the media at a concentration of 100, 50, and 25 µg/mL, respectively. All chemicals were purchased from Sigma-Aldrich.

Pre-cultures were grown overnight at 200 rpm shaking speed in 50 mL test tubes (BD Falcon) with 10 mL of M9 minimal medium supplemented with the carbon sources used in the production cultures. For inoculation of the cultures into 250 mL baffled shake flask, precultures were harvested by centrifugation for 5 min (4000×*g*, Allegra 21-R, Beckman-Coulter) and washed once with sterile distilled water. Cells were inoculated at OD_600_ ~ 0.1 into 25 mL fresh M9 minimal medium containing an appropriate concentration of carbon source (see text) and cultivated in 250 mL baffled flask on a rotary shaker (Infors HT) running at 200 rpm. Growth was followed by measure of optical density at 600 nm (OD_600_) using a Biochrom Libra S11 spectrophotometer. Expression of the GA-producing pathways was induced by addition of isopropyl β-d-1-thiogalactopyranoside (IPTG) when the OD_600_ reached ~0.8. All cultivations were carried out at 30 °C.

### Analytical methods for extracellular metabolites quantifications

Samples for metabolite quantification were regularly withdrawn from the cultures, centrifuged at 13,000 rpm for 5 min in a bench-top centrifuge (Eppendorf 5415D), filtered through a 0.2-µm syringe filter, and stored at −20 °C until further analysis. Quantification of sugars and organic acids was carried out by high performance liquid chromatography (HPLC) on an Ultimate 3000 system (Dionex, Sunnyvale, USA). The HPLC system was equipped with a cation-exchange column (Aminex HPX-87H— 300 × 7.8 mm, 9 µm, Biorad), an autosampler (WPS-3000RS, Dionex), a RI detector (RID 10A, Shimadzu), and an UV/VIS detector (SPD-20A, Shimadzu). The mobile phase was 1.25 mM H_2_SO_4_ at a flow rate of 0.5 mL/min. Column temperature was held at 35 °C.

## Results and discussion

### Metabolic engineering for optimizing glycolic acid production via the glyoxylate shunt

We first set out to engineer an *E. coli* strain for production of GA via the glyoxylate shunt by inactivating all annotated reactions that consume glyoxylic acid, i.e., malate synthase, encoded by *aceB* and *glcB* [25, 26], glyoxylate carboligase, encoded by *gcl* [27], and 2-oxo-4-hydroxyglutarate aldolase, encoded by *eda* [28, 29]. Re-oxidation of GA was prevented by deleting the glycolate oxidase-encoding *glcDEFG* operon [30]. Derepression of the glyoxylic acid-producing isocitrate lyase, AceA, was brought about by deletion of the transcriptional repressor, IclR [31, 32]. The strain which carried these deletions was transformed with plasmid pGS which expressed the isocitrate lyase and glyoxylate reductase encoding genes *aceA* and *ghrA,* respectively [33, 34] (Fig. 1). The resulting strain Pen847 produced 0.69 ± 0.23 g/l GA (0.06 g/g yield) when cultivated on mineral medium supplemented with 10 g/l glucose (Table 5). The additional deletion of the transcriptional repressor of Krebs cycle genes, ArcA [35] in strain Pen851 only slightly increased GA production to 0.80 ± 0.15 g/l (0.07 g/g yield). The isocitrate lyase from *E. coli*, AceA, has a low affinity for isocitrate (Km = 0.89 mM) when compared to isocitrate dehydrogenase (Icd, Km = 0.029 mM) [36]. Thus, it was possible that GA production was low because AceA was outcompeted by Icd. In agreement with this idea, the deletion of Icd in strain Pen1042 resulted in significant production of GA, which accumulated to 2.64 ± 0.77 g/l corresponding to a yield of 0.31 g/g (Table 5). It is of note that the strains that carried the *Δicd* deletion were unable to grow on mineral medium. To restore their growth, the cultivation medium was supplemented with yeast extract and tryptone. However, no detectable quantities of GA were produced from these supplements when no additional sugar (glucose or xylose) was provided (not shown). Significant production of GA required the overexpression of GhrA and/or AceA from plasmid pGS, since strain Pen1100 which contained the empty pACT3 plasmid did not produce any GA but accumulated nearly 5 g/l acetate (Table 5). These results are in qualitative agreement with the work of Dischert [12] and Deng [11] who reported that a strong decrease of Icd activity, brought about by decreasing the expression of *icd* or by overexpressing the Icd-inactivating protein kinase AceK, respectively, was required to achieve significant GA production.

**Table 5:** Production of glycolic acid by different *E. coli* strains in medium containing glucose as carbon source

Strain name	Additional deletions	Plasmids^a^	Biomass	Glycolic acid		Acetate	
			Yield [g/g]	Final conc. [g/l]	Yield [g/g]	Final conc. [g/l]	Yield [g/g]
Pen847		pGS	0.30 ± 0.03	0.69 ± 0.23	0.06 ± 0.01	0.06 ± 0.08	0.01 ± 0.01
Pen851	*ΔarcA*	pGS	0.30 ± 0.01	0.80 ± 0.15	0.07 ± 0.00	0.00 ± 0.00	0.00 ± 0.00
Pen1042	*ΔarcA Δicd*	pGS	0.15 ± 0.02	2.64 ± 0.77	0.31 ± 0.05	0.93 ± 0.58	0.12 ± 0.08
Pen1100	*ΔarcA Δicd*	pACT3	0.08 ± 0.00	0.00 ± 0.00	0.00 ± 0.00	4.80 ± 0.04	0.55 ± 0.00
Pen1043	*ΔarcA Δicd*	pX1P	0.12 ± 0.03	0.00 ± 0.00	0.00 ± 0.00	3.11 ± 0.96	0.47 ± 0.05
Pen1044	*ΔarcA Δicd*	pGS + pX1P	0.17 ± 0.03	3.02 ± 0.27	0.37 ± 0.03	0.22 ± 0.19	0.03 ± 0.02
Pen1048	*ΔarcA Δicd ΔxylB*	pX1P	0.09 ± 0.01	0.00 ± 0.00	0.00 ± 0.00	4.44 ± 0.13	0.61 ± 0.01
Pen905	*ΔarcA Δicd ΔxylB*	pGS + pX1P	0.16 ± 0.03	2.96 ± 0.25	0.39 ± 0.05	0.00 ± 0.00	0.00 ± 0.00

All strains carried the deletions *∆aceB ∆glcDEFGB ∆gcl ∆edd*-*eda ∆iclR.* Additional deletions are indicated in the table. Initial glucose concentration was 10 g/l. The mineral medium was supplemented with 2 g/l tryptone and 1 g/l yeast extract

^a^pGS expresses glyoxylate reductase and isocitrate lyase, encoded by *ghrA* and *aceA*. pX1P carries (d)-xylulose-1 kinase, (d)-xylulose-1 aldolase and glycolaldehyde dehydrogenase, encoded by *khkC*, *aldoB* and *aldA*, respectively. pACT3 is the empty plasmid. Results are presented as mean ± STDV calculated from at least two replicate experiments

### Co-function of glyoxylate and xylulose-1P pathways does not increase GA production on pure d-xylose

Since we wanted to quantify the increase of GA production from xylose that was due to the additional operation of the synthetic X1P pathway (see below), we first had to verify that expression of the enzymes that build up the X1P pathway have no unspecific side effects on GA production via the glyoxylate shunt. We therefore transformed plasmid pX1P, which carries the genes *khkC, aldoB,* and *aldA* genes that encode the X1P pathway enzymes, into the strains whose genotype was optimized for glyoxylate-dependent GA production and characterized them during growth on glucose. We found that the strains Pen1043 and Pen1048 which expressed pX1P alone produced no GA, thus confirming that pGS that bears GhrA and AceA genes was required for GA production through the glyoxylate shunt (Table 5). Strains Pen1044 and Pen905, which expressed both pGS and pX1P, produced GA at yields that were not statistically different from Pen1042 (Table 5). These results showed that the presence of the enzymes that build-up the X1P pathway does not significantly impact on GA production through the glyoxylate shunt. Thus, when studying GA production from xylose, which can be converted to GA through the glyoxylate and/or the X1P pathway, the observed differences could be clearly attributed to the function of the individual pathways, ruling out potential non-specific side effects of the X1P pathway enzymes.

After having demonstrated glyoxylate shunt-dependent GA production from glucose, we investigated GA production from xylose during simultaneous or individual function of the glyoxylate and X1P pathways. Strain Pen1042 assimilated xylose via the natural pentose phosphate pathway and produced GA through the glyoxylate shunt at a yield of 0.29 g/g (Table 6). Absence of GA production in the isogenic strain Pen1100, which contained the empty pACT3 plasmid instead of pGS, confirmed that overexpression of GhrA and/or AceA was also required for GA production on xylose-containing medium. To enable xylose assimilation through the synthetic X1P pathway, the xylulose-5 kinase encoding gene *xylB* was additionally deleted in the host strain which carried the engineered glyoxylate shunt. To restore growth on xylose, this strain was transformed with plasmid pX1P, and the resulting strain Pen1048 produced GA with a yield of 0.45 g/g, which corresponds to 89 % of the maximum yield (0.5 g/g) of the synthetic pathway. The GA yield of Pen1048 was 55 % higher than for Pen1042, indicating that GA production by the X1P pathway was more efficient than by the glyoxylate shunt. Contrary to our expectation, the simultaneous operation of the glyoxylate and X1P pathways in strain Pen905 did not result in a further increase of the GA yield which only reached 0.43 g/g (Table 6). The reason for the failure to increase GA production by the co-function of both pathways during growth on pure xylose is not entirely clear. We speculate that the metabolic burden due to the propagation of two plasmids and the severely reduced Krebs cycle function due to the deletion of *icd* were responsible for the very strong growth retardation of this strain (not shown) which ultimately impaired efficient GA production under these conditions.

**Table 6:** Production of glycolic acid by different *E. coli* strains in medium containing xylose as carbon source

Strain name	Additional deletions	Plasmids^a^	Biomass	Glycolic acid		Acetate	
			Yield [g/g]	Final conc. [g/l]	Yield [g/g]	Final conc. [g/l]	Yield [g/g]
Pen1100		pACT3	0.10 ± 0.00	0.00 ± 0.00	0.00 ± 0.00	4.20 ± 0.01	0.57 ± 0.00
Pen1042		pGS	0.16 ± 0.01	2.11 ± 0.49	0.29 ± 0.05	0.16 ± 0.23	0.02 ± 0.03
Pen1044		pGS + pX1P	0.13 ± 0.03	1.57 ± 0.24	0.24 ± 0.02	0.55 ± 0.11	0.08 ± 0.02
Pen1048	*ΔxylB*	pX1P	0.09 ± 0.01	2.70 ± 0.16	0.45 ± 0.01	1.97 ± 0.31	0.33 ± 0.04
Pen905	*ΔxylB*	pGS + pX1P	0.08 ± 0.01	2.24 ± 0.46	0.43 ± 0.05	1.34 ± 0.01	0.26 ± 0.02

All strains carried the deletions *∆aceB ∆glcDEFGB ∆gcl ∆edd*-*eda ΔiclR ΔarcA Δicd.* Additional deletions are indicated in the table. Initial xylose concentration was 10 g/l. The mineral medium was supplemented with 2 g/l tryptone and 1 g/l yeast extract

^a^pGS expresses glyoxylate reductase and isocitrate lyase, encoded by *ghrA* and *aceA*. pX1P carries (d)-xylulose-1 kinase, (d)-xylulose-1 aldolase and glycolaldehyde dehydrogenase, encoded by *khkC*, *aldoB* and *aldA*, respectively. pACT3 is the empty plasmid. Results are presented as mean ± STDV calculated from at least two replicate experiments

### Simultaneous function of the glyoxylate and (d)-xylulose-1P glycolic acid pathways strongly increases product yield during co-assimilation of glucose and xylose

**Fig. 2:**
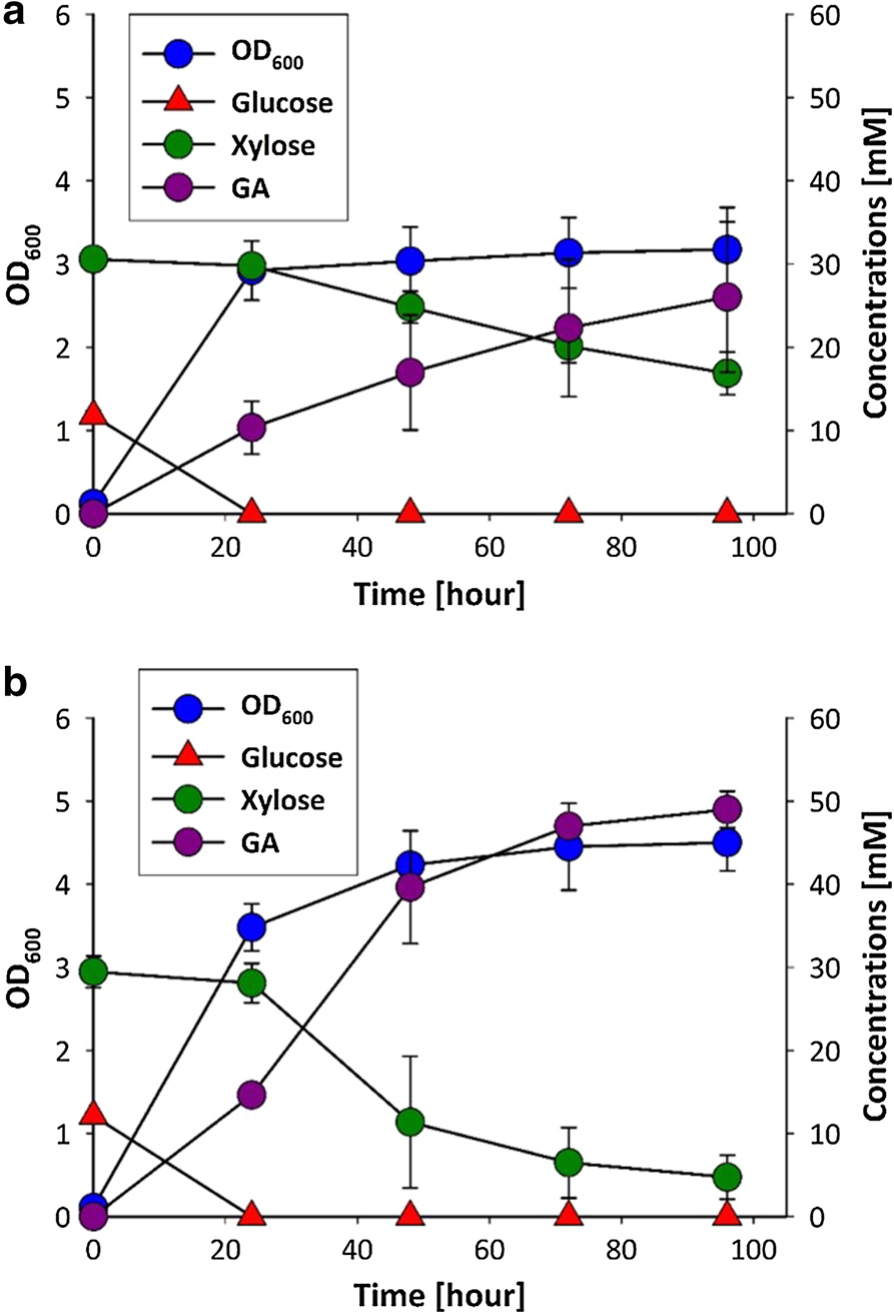
Production of glycolic acid (GA) by optimized *E. coli* strains during growth on a synthetic mixture. **a** Strain Pen905 (*∆aceB ∆glcDEFGB ∆gcl ∆edd*-*eda ∆iclR ∆arcA ∆icd ∆xylB* expressing pGS and pX1P). **b** Strain Pen979 (*∆aceB ∆glcDEFGB ∆gcl ∆edd*-*eda ∆iclR ∆arcA ∆icd ∆xylB* *galP* ^*proD*^ expressing pGS and pX1P). Initial glucose and xylose concentrations were 2.5 and 5 g/l, respectively. Cultivation was carried out in shake flasks at 30 °C

The development of the synthetic X1P pathway and its simultaneous operation with the engineered glyoxylate shunt was meant to increase GA production from xylose-rich second generation feedstocks. Hemicellulosic hydrolysates typically contain 50–75 % xylose and 15–40 % glucose together with smaller amounts of other sugars [17, 37]. In order to test whether GA production from these feedstocks could be increased by the simultaneous operation of the X1P and glyoxylate pathways, we chose to incubate our strains on a sugar mixture that contained 2.5 g/l glucose and 5 g/l xylose, which corresponds to a mass ratio (33 %/66 %) that is representative for hemicellulosic hydrolysates. Strains Pen1042 and Pen1044, which produce GA exclusively through the glyoxylate shunt, exhibited very similar GA yields of 0.37 and 0.42 g/g, respectively, that were calculated based on the total amounts of consumed sugars (glucose+xylose, Table 7). Due to the sequential utilization of glucose and xylose by these strains (not shown), the GA yield on the corresponding sugar fractions could be calculated individually. Interestingly, the GA yield on the xylose fraction of the sugar mixture (0.47 g/g) was significantly higher than when these strains were incubated on pure xylose (0.29 g/g). The reason for this behavior is not entirely clear, but it appears likely that more xylose could be converted to GA due to the very residual growth during utilization of the xylose fraction of the sugar mixture (compare to Fig. 2).

**Table 7:** Production of glycolic acid (GA) by different *E. coli* strains in medium containing glucose and xylose as carbon sources

Strain name	Additional modifications	Plasmids^a^	Biomass	GA	GA yield on consumed sugar^b^		
			Yield [g/g]	Final conc. [g/l]	G + X [g/g]	G [g/g]	X [g/g]
Pen1042		pGS	0.30 ± 0.01	1.58 ± 0.11	0.37 ± 0.04	0.30 ± 0.05	0.47 ± 0.04
Pen1044		pGS + pX1P	0.26 ± 0.03	2.41 ± 0.64	0.42 ± 0.01	0.36 ± 0.09	0.49 ± 0.04
Pen1099	*galP* ^*proD*^	pGS + pX1P	0.24 ± 0.00	1.29 ± 0.00	0.33 ± 0.00	0.36 ± 0.02	0.35 ± 0.02
Pen905	*∆xylB*	pGS + pX1P	0.22 ± 0.02	1.98 ± 0.68	0.47 ± 0.12	0.37 ± 0.12	0.60 ± 0.11
Pen979	*∆xylB galP* ^*proD*^	pGS + pX1P	0.23 ± 0.02	3.73 ± 0.16	0.63 ± 0.04	0.40 ± 0.01	0.75 ± 0.05

All strains carried the deletions *∆aceB ∆glcDEFGB ∆gcl ∆edd*-*eda ΔiclR ΔarcA Δicd.* Additional modifications are indicated in the table. Initial glucose and xylose concentrations were 2.5  and 5 g/l, respectively. The mineral medium was supplemented with 2 g/l tryptone and 1 g/l yeast extract

*G* glucose, *X* xylose, *G* *+* *X* total sugar

^a^ pGS expresses glyoxylate reductase and isocitrate lyase, encoded by *ghrA* and *aceA*. pX1P carries (d)-xylulose-1 kinase, (d)-xylulose-1 aldolase and glycolaldehyde dehydrogenase, encoded by *khkC*, *aldoB* and *aldA*, respectively. pACT3 is the empty plasmid

^b^ Results are presented as mean ± STDV calculated from at least two replicate experiments

Strain Pen905, which operates both the glyoxylate and the X1P pathway, produced GA with a yield of 0.47 g/g. Thus, the simultaneous operation of the X1P and glyoxylate pathways conferred a ~27 % increased GA yield when compared to the exclusive operation of the glyoxylate pathway. The GA yield on the xylose fraction was 0.6 g/g (Table 7) which is significantly higher than the GA yield which was reached by this strain on pure xylose (0.43 g/g, Table 6). The simplest way to explain this difference is that the GA-producing pathways could be appropriately expressed in the sugar mixture during the exponential growth phase on glucose, whereas GA production on pure xylose remained comparatively low because growth of the cells was extremely retarded resulting in incomplete expression of the GA pathways.

As shown in Fig. 2a, strain Pen905 first consumed all glucose in the medium before starting to utilize xylose. No growth was observed after glucose had been depleted and even after 100 h of cultivation the strain was only capable of consuming ~50 % of the xylose fraction. It was previously reported that stationary *E. coli* cells have a strongly decreased glucose uptake rate compared to exponentially growing cells [38, 39]. Our results suggest that xylose uptake is also reduced in the absence of growth. In an attempt to facilitate co-assimilation of glucose and xylose and/or to increase the xylose uptake rate during stationary phase, we replaced the natural promoter of the broad-range sugar permease, GalP [40, 41], by the strong constitutive promoter proD [22]. The resulting strain Pen979 continued to consume glucose and xylose sequentially, but exhibited strongly improved sugar uptake rates and consumed nearly all xylose during the monitored incubation period (Fig. 2b). As a consequence, the GA yield of strain Pen979 on total consumed sugar increased to 0.63 g/g, which corresponds to a gain of ~70 % compared to strain Pen1042 which operates the glyoxylate pathway alone. The GA yield on the xylose fraction reached 0.75 g/g (Table 7). This value corresponds to 75 % of the theoretical maximum GA yield which can be achieved during simultaneous operation of the glyoxylate and X1P pathways, and is the highest GA yield reported so far.

In contrast to the Pereira et al. [14], who reported a GA yield of 0.63 g/g on pure xylose (Table 1), we chose to delete *icd* in our GA-producing strains, which resulted in the complete inactivation of the oxidative Krebs cycle branch thus increasing the carbon flux into the GA-producing glyoxylate shunt. This metabolic engineering strategy proved very effective for increasing the GA yield, but also imposed the need for supplementing the cultivation medium with an amino acid source (yeast extract and tryptone in the present study) to enable growth of our strains. It remains to be evaluated whether the need for amino acid supplements can be tolerated in an industrial process.

## Conclusions

We have demonstrated that the simultaneous operation of the synthetic X1P and the engineered glyoxylate pathways greatly increases the GA yield on xylose-rich sugar mixtures. These results confirm the strong stoichiometric advantage that is provided by the synthetic X1P pathway during GA production from xylose containing feedstocks. However, further strain optimization is required to improve growth and fermentation characteristics of the production strains. It can be expected that genomic integration of the genes that are currently expressed from the pGS and pX1P plasmids will alleviate a significant metabolic burden, thus rendering growth of the cells more robust. In addition, our approach to enable GA production though the glyoxylate shunt by deleting *icd* strongly impaired growth of the cells. Thus, a more elaborate attenuation of Icd activity that maintains the ability of the cells to grow on mineral medium, e.g. by reducing its expression or by overexpressing *aceK* [12, 14], is clearly preferable when developing a strain for industrial applications. On the other hand, we reached a very high GA yield of 0.75 g/g during the utilization of the xylose fraction of the sugar mixture. This shows that preventing growth during xylose utilization may be a promising approach to make full use of the stoichiometric advantage that is provided by the simultaneous operation of the glyoxylate and X1P pathways.

## Abbreviations

GA: glycolic acid
HPLC: high performance liquid chromatography
IPTG: isopropyl β-d-1-thiogalactopyranoside
OD_600_: optical density at 600 nm
PGA: poly-glycolic acid
pGS: plasmid which express the isocitrate lyase (*aceA*) and glyoxylate reductase (*ghrA*)encoding genes
pX1P: plasmid which express the ketohexokinase C (*khkC*), aldolase B (*aldoB*) and aldehyde dehydrogenase (*aldA*) encoding genes
RI: refractive index
UV/VIS: ultraviolet–visible
X1P: (d)-xylulose-1 phosphate
